# Exosome-Derived microRNA: Potential Target for Diagnosis and Treatment of Sepsis

**DOI:** 10.1155/2024/4481452

**Published:** 2024-07-25

**Authors:** Yujie Xiao, Yixuan Yuan, Dahai Hu, Hongtao Wang

**Affiliations:** Department of Burns and Cutaneous Surgery Xijing Hospital Fourth Military Medical University, 127 West Chang-le Road, Xi'an 710032, Shaanxi, China

## Abstract

Exosome-derived microRNAs (miRNAs) are emerging as pivotal players in the pathophysiology of sepsis, representing a new frontier in both the diagnosis and treatment of this complex condition. Sepsis, a severe systemic response to infection, involves intricate immune and nonimmune mechanisms, where exosome-mediated communication can significantly influence disease progression and outcomes. During the progress of sepsis, the miRNA profile of exosomes undergoes notable alterations, is reflecting, and may affect the progression of the disease. This review comprehensively explores the biology of exosome-derived miRNAs, which originate from both immune cells (such as macrophages and dendritic cells) and nonimmune cells (such as endothelial and epithelial cells) and play a dynamic role in modulating pathways that affect the course of sepsis, including those related to inflammation, immune response, cell survival, and apoptosis. Taking into account these dynamic changes, we further discuss the potential of exosome-derived miRNAs as biomarkers for the early detection and prognosis of sepsis and advantages over traditional biomarkers due to their stability and specificity. Furthermore, this review evaluates exosome-based therapeutic miRNA delivery systems in sepsis, which may pave the way for targeted modulation of the septic response and personalized treatment options.

## 1. Introduction

**Figure 1:**
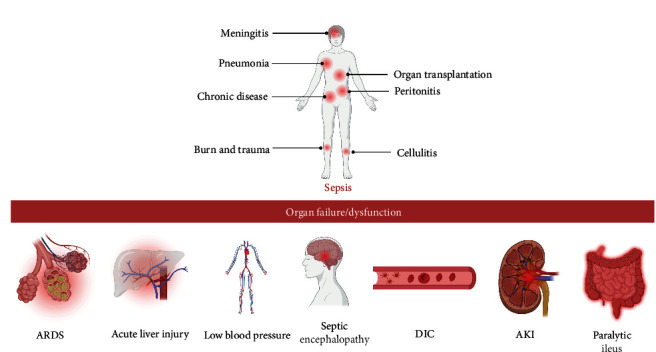
Sepsis is a critical clinical syndrome arising from the host's dysregulated response to infection, which can have a profound impact on multiple physiological systems and organs. If not addressed promptly, this pathological reaction can escalate to organ dysfunction and potentially lead to multi-organ failure. The foundation of effective sepsis management is rapid and accurate diagnosis, followed by targeted therapeutic interventions. In parallel, it is essential to implement robust organ support measures designed to dampen the systemic inflammatory response and preserve organ function. Created with BioRender.com.

Sepsis is a life-threatening organ dysfunction caused by the host's dysregulation of infection response [1]. As the primary cause of mortality in intensive care units [2], sepsis, with controversial diagnostic criteria [3], is predominantly managed through supportive and symptomatic treatments in the absence of specific drugs [4]. The pathogenesis of sepsis primarily involves pathogen-associated molecular patterns (PAMPs) and damage-associated molecular patterns (DAMPs). PAMPs and DAMPs are identified by pattern recognition receptors (PRRs) in immune cells, including Toll-like receptors (TLRs) [5]. PAMPs are structures inherently present on microorganisms [6], whereas DAMPs are nonmicrobial molecules released following tissue damage [5]. When PAMPs and DAMPs are recognized by innate immune cells, intracellular signalling pathways are activated, enabling interactions among various immune cells (neutrophils, monocytes, macrophages, dendritic cells, and lymphocytes) through these signals. These pathways collaborate to initiate immune responses for self-protection [7]. In sepsis, certain signalling pathways can become overactivated, leading to the overproduction of DAMPs, various inflammatory mediators, and cytokines, culminating in a cytokine storm. This may cause abnormal immune cell regulation, leading to systemic inflammation and disordered immune responses. Excessive inflammatory responses can result in cellular and tissue damage, and eventually molecular dysfunction, culminating in organ failure [7, 8]. Given the multifaceted etiology of sepsis, its variable clinical symptoms, and rapid progression to death, it is particularly urgent to search for earlier, more sensitive, and specific biomarkers that rapidly and precisely identify early sepsis and prognosis, as well as novel therapeutic targets (Figure 1).

Exosomes are single-membrane extracellular vehicles (EVs) released by the endosomal system, which play a crucial role in cellular trafficking and signalling under physiological and pathological conditions [9, 10]. Exosomes, which represent a subtype of small EVs typically smaller than 200 nm, are derived from outward growth of the plasma membrane or inward growth that forms multivesicular bodies (MVB) and subsequently fuse with the plasma membrane [10]. The major bioactive components present in exosomes comprise proteins, lipids, mRNA, and microRNA (miRNA). These components are enriched not only with substances derived from the origin cells, which reflects their heterogeneity, but also facilitate communication between the original cells and target cells [10, 11]. This makes exosomes fundamental regulators of intercellular signalling, which play a key role in mediating inflammatory processes. At the same time, conditions such as oxidative stress, endoplasmic reticulum stress, ribosomal stress, thermal stress, infection, inflammation, and injury can alter the concentration and contents of exosomes. Consequently, the analysis of the contents of exosomes provides valuable information on the disease state of tissues [12, 13, 14, 15]. Exosomes, as critical tools for intercellular communication, carry a wealth of genetic information, including messenger RNA (mRNA) and microRNA (miRNA). During the process of information transfer by exosomes, although most RNAs are degraded into fragments shorter than 200 nucleotides, some intact RNAs can be absorbed by other cells through endocytosis, thus affecting protein synthesis in these target cells. These intact RNAs may have significant potential for disease diagnosis and treatment. For example, in sepsis research, it has been discovered that miRNAs within exosomes are associated with the severity of organ failure and mortality in patients, indicating that exosomes play a regulatory role in the pathophysiological processes of sepsis by carrying miRNAs. The miRNAs released by exosomes can propagate between cells, influencing gene expression and protein synthesis in the recipient cells, thus significantly affecting inflammation and immune responses. Therefore, exosomes and their RNA components are increasingly recognized to play a vital role in disease diagnosis and therapy [16, 17, 18, 19]. Furthermore, exosomes have been used to differentiate sepsis from noninfectious systemic inflammation to improve the diagnostic precision of sepsis, suggesting that exosomal miRNAs are emerging as promising diagnostic biomarkers [20].

This article reviews the progress of research on the role of miRNA in exosomes from diverse sources in sepsis development and discusses the potential of exosomal miRNA in its diagnosis and treatment. Considering the crucial role of immune cells in the pathogenesis of sepsis, this review focuses on immune cells including neutrophils, platelets, macrophages, and endothelial cells. Exosomes from these cells constitute the primary source of host-derived exosomes in sepsis [21, 22, 23, 24, 25]. The presence of miRNA-containing exosomes in bodily fluids such as blood and urine underscores their potential as novel biomarkers [26, 27, 28, 29]. Investigations into exosomal miRNAs and their roles in disease mechanisms offer fresh insights into the diagnosis and treatment of sepsis. Certain exosomal miRNAs are crucial in sepsis progression and are expected to become effective diagnostic and therapeutic tools.

## 2. The Diagnostic Potential of Exosomal miRNAs in Sepsis

### 2.1. Exosomal miRNAs as Sepsis Biomarkers

**Figure 2:**
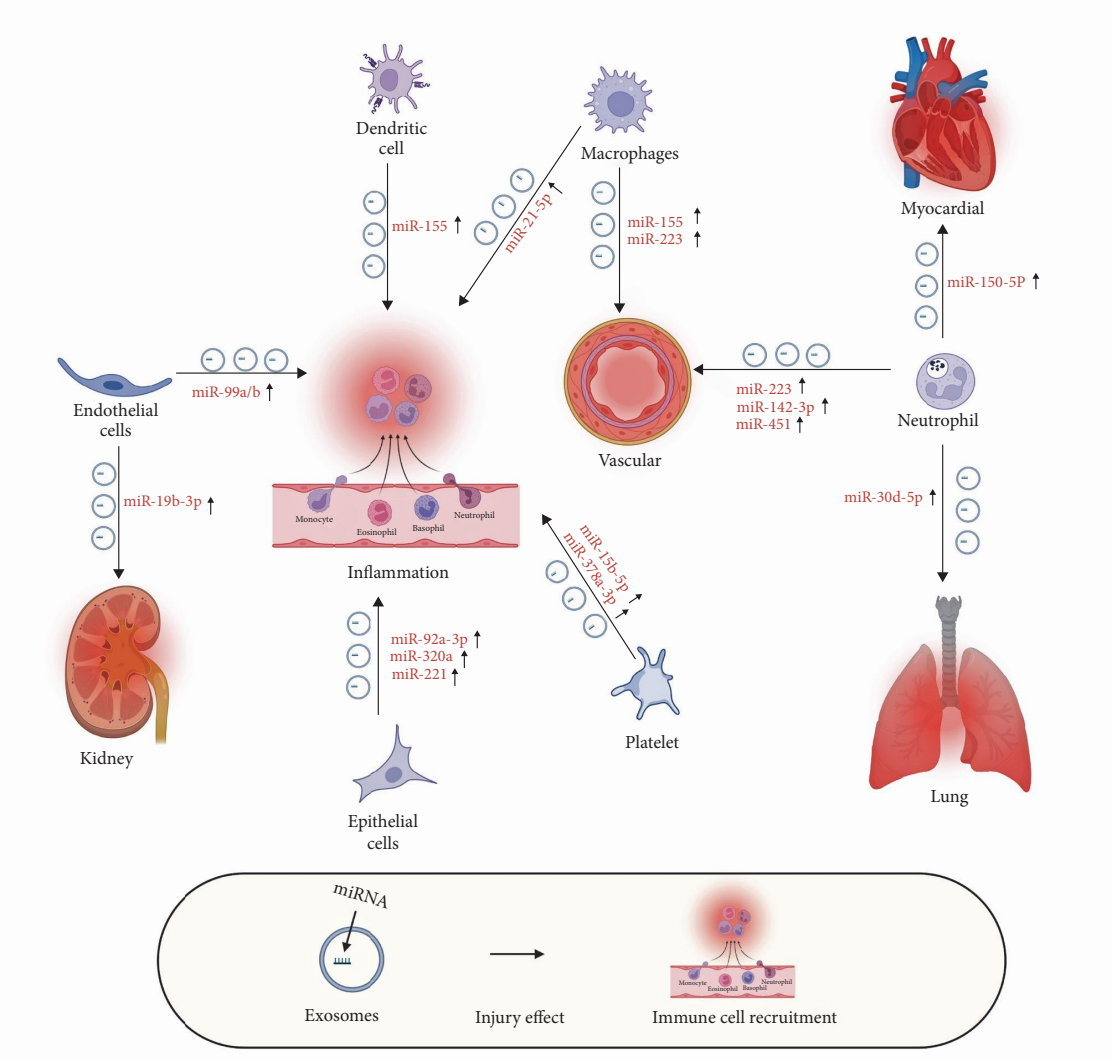
The role of exosomal miRNA secreted by various cells in organ dysfunction during sepsis. Exosomal miRNA can be upregulated, thus playing a crucial role in the onset and progression of sepsis. These miRNAs are secreted by various immune cells, including macrophages, neutrophils, and dendritic cells, along with nonimmune cells such as platelets and endothelial cells, and contribute to various inflammatory reactions, playing a key role in organ dysfunction during sepsis. Created with BioRender.com.

A significant advantage of exosomes is that they contain the status and function of their source cells, thus playing a crucial role in transmitting cellular information. Furthermore, the unique surface molecules and exosome content dictate their ability to influence the pathophysiological processes of various diseases, making them extensively used in biomarker research for diverse diseases [30, 31, 32]. In particular, miRNA within exosomes has garnered significant attention in the realm of sepsis as biomarkers. Research indicates a close correlation between the expression pattern of exosomal miRNA in patients with sepsis and their progression of the disease. Recent studies have highlighted the significant role of specific miRNAs in the early diagnosis, management, and prognosis of sepsis [33, 34, 35, 36]. These miRNAs serve as crucial biomarkers, reflecting the severity of inflammation, the status of the immune system, and the extent of cellular damage, thus providing invaluable information for the diagnosis of sepsis. Their altered expression patterns offer a promising avenue for the development of more targeted diagnostic and therapeutic strategies [37, 38, 39]. In particular, research has revealed that in patients with sepsis, the levels of miRNAs related to antioxidant defense and oxidative stress are variably altered within exosomal vesicles. This variation suggests a potential application of these miRNAs as biomarkers to accurately distinguish sepsis cases. Such findings underscore the importance of miRNAs in the pathophysiology of sepsis and their potential to improve the precision of clinical interventions [40] (Figure 2).

### 2.2. Exosomal miRNA Derived from Neutrophils

Neutrophils, as the primary white blood cells recruited to infection sites during sepsis, are crucial innate immune cells that contribute to host damage [41]. In different stages of sepsis, the immune response and neutrophil activity show significant variations, leading to considerable differences in the secretion of exosomes and their corresponding cargo [42]. In the initial phase of sepsis, neutrophils undergo excessive activation, resulting in a continuous increase in exosome secretion as sepsis progresses. These exosomes may contain an abundance of pro-inflammatory miRNAs and proteins, which contribute to the amplification and exacerbation of the inflammatory response [43]. In the later stages of sepsis, the function of neutrophils can be altered, and the immune system suppressed, making patients more susceptible to secondary infections. Neutrophils in this phase release exosomes that contain components associated with immune suppression, such as regulatory T cell-related miRNAs and proteins, making patients more susceptible to secondary infections during the potential secondary infection phase. Exosomes released during this stage may contain signalling molecules related to cellular functional inhibition and immune exhaustion, leading to reduced neutrophil activity, regulation of excessive immune responses, and potentially reducing tissue damage [44]. This underscores the substantial diagnostic potential of the content within neutrophil-derived exosomes at various stages of sepsis [42, 45, 46]. Additionally, it was documented that the exosomes secreted exhibit abnormal levels of RNA expression at different stages of sepsis, exacerbating or inhibiting the inflammatory response, indicating that neutrophil exosome RNA can serve as a potential diagnostic marker for sepsis [43, 47]. For example, activated neutrophils secrete exosomes in response to inflammatory stimuli, including upregulated miR-223, miR-142-3p, and miR-451, that initiate an inflammatory cascade and result in direct vascular damage [47]. Exosomal miR-30d-5p, originating from polymorphonuclear neutrophils (PMN), targets SOCS-1 and SIRT1, upregulates NF-*κ*B signalling, which induces macrophage M1 polarization and initiates macrophage pyroptosis. This process aggravates the inflammatory response and bodily damage associated with sepsis and leads to acute lung injury, and upregulated miR-30d-5p is considered to possess diagnostic value [48]. The presence of miR-150-5P in neutrophil-derived exosomes is instrumental in the progression of sepsis to cardiomyopathy and is recognized as a potential predictor of both the deterioration of sepsis and its progression to cardiomyopathy [49].

### 2.3. Exosomal miRNA Derived from Monocytes and Macrophages

Macrophages, as professional antigen-presenting cells (APCs), constitute the first-line of defense of the human immune system and play a crucial role in immune suppression during sepsis [50, 51]. PRRs identify PAMPs or DAMPs, thus initiating complex metabolic cascade reactions [51]. Simultaneously, pathogens are phagocytosed and presented to helper T cells (Th) [52]. Macrophages may polarize into M1 macrophages, which release pro-inflammatory factors, or M2 macrophages, which release anti-inflammatory factors, based on the microenvironment [53, 54]. The progression of sepsis may be exacerbated by an imbalance between M1 and M2 macrophages. In particular in the early stages of sepsis, there may be a tendency towards a shift to M1-type macrophages, while in later stages, a transition to M2-type may occur, depending on dynamic changes in the immune response [55]. Along with changes in the polarization state of macrophages, the expression patterns of miRNAs in exosomes associated with polarization can also change. Monitoring macrophage-secreted exosomes and their miRNA content could potentially aid in the early diagnosis and treatment of sepsis. Research has shown that macrophage-derived exosomes are upregulated in response to inflammatory stimulation or stress [56]. Another study has identified that macrophage-derived exosomes lead to glomerular endothelial cell dysfunction and acute kidney injury, highlighting the importance of macrophage-derived exosomes in sepsis diagnosis [57]. In the context of sepsis, the significance of macrophage-derived exosomal miRNA is evident, as it is markedly associated with disease severity, progression, and patient prognosis, simultaneously exerting anti-inflammatory effects and contributing to homeostasis regulation [57]. In particular, exosomes derived from M1 macrophages and miRNAs they encapsulate are recognized for their role in promoting tissue damage and organ dysfunction by mediating signalling pathways between immune cells and target cells. This dual impact underscores the complex interplay between macrophage-derived exosomal miRNA and the pathophysiological processes observed in sepsis, revealing potential avenues for therapeutic intervention and a deeper understanding of the mechanisms of the disease [58]. LPS-stimulated macrophages secrete exosomes containing elevated levels of miR-21-3p, miR-146a, and miR-146b, which are known to prevent overactivation of innate immune responses. As a result, these miRNAs could potentially serve as biomarkers for sepsis, contributing to early diagnosis and disease monitoring [59, 60]. M2 macrophage-derived exosomes specifically target and negatively regulate HMGB1 expression through miR-216a, thus downregulating the TLR4-NF-*κ*B signalling pathway and producing antinociceptive effects in CIBP mouse models. MiR-216a, linked to immune suppression and inflammation regulation, has potential as an indicator for sepsis diagnosis [61]. The upregulated miR-24-3p in macrophage-derived M2 exosomes improves cardioprotection after myocardial injury in sepsis by decreasing Tnfsf10 expression, positioning miR-24-3p as a potential sepsis biomarker [62]. Increased levels of miR-155 and miR-223 in monocyte-derived exosomes upregulate the release of inflammatory factors by the TLR4/NF-*κ*B signalling pathway in endothelial cells, resulting in subsequent inflammatory and endothelial damage [63]. Moreover, a study on diabetic nephropathy highlighted the importance of miR-21-5p in macrophage-derived exosomes for inflammation regulation, applicable not only to specific diseases such as diabetic nephropathy but also to sepsis, indicating the potential role of miR-21-5p in the early diagnosis of sepsis [64].

### 2.4. Exosomal miRNA Derived from Dendritic Cells

Dendritic cells (DCs), which function as APCs, are essential to initiate protective immunity in the early stages of inflammation. They not only recognize and respond to invading microorganisms, but also deliver phagocytosed pathogen antigens to T cells in the later stages of adaptive immunity. This function is vital in maintaining immune balance by bridging innate and adaptive immunity [65, 66]. Studies show that decreases in the number of DCs, increase in monocytes differentiating into DCs, and changes in the levels of surface molecules related to DC function [67, 68, 69] are considered potential targets for sepsis diagnosis, and reducing DC autophagy is proposed as a novel and effective strategy to mitigate sepsis [70]. Exosomes derived from DCs demonstrate potential in the early diagnosis and immunotherapy of sepsis, surpassing cells themselves. Evidence indicates that DC-derived exosomes have the ability to internalize and transfer antigenic peptides to MHC molecules from nearby DCs, facilitating the transport of these MHC/peptide complexes to the surface of DC for presentation to T cells [71, 72]. Initial research revealed that EGF factor VIII (MFG-E8) containing milk fat globule in immature DC-derived exosomes enables recognition of apoptotic immune cells by phagocytes in sepsis. This mechanism helps to attenuate the systemic inflammatory response and reduce the overall damage associated with sepsis [73]. Other studies uncovered that in exosomes of bone marrow-derived DCs, miR-155 is upregulated to intensify the inflammatory response in sepsis, while miR-146a is downregulated to suppress it [74]. Furthermore, another study showed that exosomes of these DCs carry miR-146a, which is involved in a negative feedback loop regulating inflammation [75]. Therefore, the contents of these exosomes possess significant potential for diagnosis of sepsis, although research exploring the relationship between DC-derived exosomes and sepsis remains in the early stages.

### 2.5. Exosomal miRNA Derived from T Cells

T cells, including CD4 T cells, play a crucial role in the development of cellular and humoral immune responses after infection. Throughout different stages of sepsis, there are notable variations in the expression of exosomes from Th and regulatory T cells (Treg). These changes exert significant regulatory effects during the progression of sepsis [76]. Understanding alterations in T cell subpopulations and their exosomes is crucial for enhancing diagnostic accuracy and formulating effective therapeutic strategies in the diagnosis and treatment of sepsis. Research indicates that Treg implements peripheral tolerance through various mechanisms, reflecting the complexity and adaptability of the immune response. Treg-derived exosomes can play a role in maintaining self-tolerance and restricting other immune reactions, which hold potential value for therapeutic strategies in sepsis [77]. A study suggests that miR-150-5p and miR-142-3p, which are contained within Treg-secreted exosomes, become upregulated and are subsequently transferred to endothelial cells, thus enhancing endothelial cell activation during the immune response [78]. This observation suggests the existence of a reciprocal activation mechanism between T lymphocytes and dendritic cells as well as their diagnostic and therapeutic value in sepsis. Additionally, sepsis can induce changes in the immune system, manifesting itself notably as a decrease in the quantity and functionality of lymphocytes. This condition, known as immune paralysis [79], is characterized by unique alterations in EV secreted by lymphocytes. Consequently, understanding the roles of T cell subpopulations and their extracellular vesicle content in sepsis is essential not only to elucidate the immunological mechanisms underlying the disease but also to potentially offer new directions for the diagnosis and treatment of sepsis. However, the investigation of lymphocyte-derived exosomes remains in an exploratory stage. The potential role of lymphocyte-derived exosomes in the future of sepsis diagnosis or treatment has not yet been definitively determined.

### 2.6. Exosomal miRNA Derived from Mesenchymal Stem Cells

Mesenchymal stem cells (MSCs), primarily derived from embryonic mesoderm and ectoderm embryonic tissues [80], have the capacity to regulate both innate and adaptive immune responses, particularly by regulating the proliferation and migration of CD4+, CD8+ T, and NK cells [81, 82]. In sepsis, exosomes from MSCs manifest a remote immunomodulatory effect, influencing the interaction between MSCs and immune cells and contributing to the regulation of inflammatory responses. This modulation has the potential to alter the characteristics of specific immune cells, aiding in the control of excessive inflammatory activity. Therefore, MSC-derived exosomes (MSC-Exos) could play a crucial role in the treatment of sepsis, offering novel therapeutic strategies [81, 83, 84]. Research has shown that MSC-Exos are capable of directly presenting antigenic motifs to immune cells, thus activating CD8+ T cells and NK cells [85]. In recent years, the role of Adipose-derived stem cell derived exosomes (ADSC-Exo) in modulating immune responses, especially in the context of sepsis, has been increasingly emphasized. These exosomes are capable of inducing macrophage polarization toward an anti-inflammatory phenotype M2 through the regulation of Nrf2 and HO-1 expression, thus ameliorating inflammation and multi-organ damage in sepsis [86]. Furthermore, the mitochondrial delivery system of these exosomes selectively transports circRNA mSCAR into macrophage mitochondria, facilitating polarization towards the M2 subtype [87]. ADSC-Exo have been shown to alleviate sepsis-induced acute lung injury (ALI) by promoting macrophages to secrete TGF-*β* [88], highlighting their potential as a treatment for sepsis. Due to the key role of miRNA in mesenchymal stem cell-derived exosomes in regulating gene expression and orchestrating immune and inflammatory responses, miRNA expression patterns undergo considerable changes, potentially serving as targets for early diagnosis and treatment. For example, miRNA-191 in exosomes derived from bone MSCs is found to be upregulated, leading to inhibition of the expression of the 3′-UTR of DAPK1 mRNA in THP-1 macrophages, thus curbing LPS-induced inflammatory activation [89]. Similarly, miR-21a-5p in bone MSC-Exos targets TLR 4 and programmed cell death 4, effectively attenuating inflammation and mitigating sepsis. This indicates that MSC-Exos carrying miR-21a-5p may represent a promising strategy for the diagnosis and treating sepsis [90]. While the therapeutic potential of MSCs is well documented, their diagnostic value in sepsis requires additional exploration and validation.

### 2.7. Exosomal miRNA Derived from Endothelial Cells

Endothelial cell-derived exosomes are crucial in various physiological processes. Serving as a critical interface between circulating blood and parenchymal cells, endothelial cells are responsible for maintaining the integrity of the vascular barrier, regulating inflammation, facilitating cell signalling, and supporting hemostatic functions [91, 92]. Endothelial cells are among the first to detect endogenous metabolites in the blood, serving as sensors for danger signals and foreign microorganisms [93]. In the early stage of sepsis, endothelial cells become hyperactivated, resulting in a compromise in their barrier and anti-inflammatory capabilities [94, 95]. This state further promotes the recruitment of inflammatory mediators and immune cells, including the release of various exosomes, thereby establishing intricate connections [94, 95, 96, 97]. In addition to an increase in the number of exosomes secreted by endothelial cells [97, 98], studies have also shown that endothelial cell-derived exosomes can enhance the pro-inflammatory response in monocytes by upregulating miR-99a/b and targeting mTOR expression [99], emphasizing the diagnostic relevance of miRNA within exosomes. In addition to their role in exacerbating damage, certain miRNAs in exosomes are capable of mitigating sepsis-induced damage through regulatory mechanisms, offering diagnostic and therapeutic possibilities. For example, miR-125b-5p, which is upregulated in endothelial cell exosomes, inhibits TOP2A expression, thus attenuating acute lung injury induced by sepsis [100]. Likewise, the upregulation of miR-21-5p results in the downregulation of RUNX1, thereby reducing renal injury from sepsis [101]. Additionally, the elevation of miR-382-3p in endothelial cells, which targets BTRC expression, leads to a reduction in phosphorylation of I*κ*B*α*/NF-*κ*B, thereby alleviating organ damage and immunosuppression in sepsis [102]. Significantly, exosomes from endothelial progenitor cells transport miR-126 to enhance recovery from acute lung injury caused by sepsis [88].

### 2.8. Exosomal miRNA Derived from Epithelial Cells

Epithelial cells, located at the environmental interface, regularly encounter pathogenic microorganisms. These cells are skilled in detecting changes in the microenvironment and transmitting signals to immune cells, thus initiating inflammatory responses [103]. The exact mechanisms by which epithelial cells communicate with various types of immune cells are not fully understood; however, current research suggests that epithelial cells play a role in intercellular communication by regulating the content of exosomes, especially exosome miRNAs [104, 105]. As a result, exosomal miRNAs from epithelial cells possess significant potential to diagnose and treat sepsis. For example, miR-92a-3p levels are found to be elevated in exosomes secreted by lung epithelial cells during septic lung injury, resulting in reduced expression of the PTEN protein in macrophages, thus activating alveolar macrophages and exacerbating ischemia-reperfusion-induced inflammation [106]. Another study identified that miR-155 in exosomes, partially derived from lung epithelial cells, promotes macrophage proliferation by targeting SHIP1 and SOCS1, aggravating septic lung injury [16]. Under stress conditions, both miR-320a and miR-221 experience upregulation in epithelial cell exosomes, thus further enhancing macrophage-mediated inflammatory responses [107]. In contrast, miR-19b-3p upregulation in renal epithelial cell-derived exosomes results in increased activation of M1 type macrophages, exacerbating renal injury [20]. Alternatively, a decrease in miR-145 in lung epithelial cell-derived exosomes, which targets TGFBR2, leads to the inactivation of TGFBR2/Smad3 signalling, thus inhibiting lung injury induced by sepsis [108]. These findings emphasize the significant role of epithelial cell-derived exosomes and their miRNA content in modulating immune responses and sepsis, indicating their potential as biomarkers for diagnosis and as therapeutic targets.

### 2.9. Exosomal miRNA Derived from Platelets

Historically, platelets have been predominantly associated with coagulation, the formation of hemostatic thrombi, and the facilitation of contraction. However, recent advances in platelet research have revealed their more complex roles, particularly in the context of sepsis. Inflammation-induced platelet aggregation can further recruit and activate innate immune cells, thus exacerbating inflammation and disseminated intravascular coagulation, leading to organ dysfunction during sepsis [109, 110, 111, 112, 113]. Recent studies have demonstrated that platelet-derived exosomes are vital in neutrophil extracellular trap formation (NET), facilitating neutrophil activation and adhesion, and amplifying the inflammatory response after tissue injury, potentially precipitating the onset of sepsis [114, 115, 116]. Additionally, during sepsis, exosomes are known to induce myocardial dysfunction [117]. Significantly, platelet exosome production increases substantially during sepsis [118, 119], inhibitors of IKK (I*κ*B kinase) can reduce lung injury in sepsis by suppressing the secretion of platelet-derived exosomes [120], which opening up new avenues for sepsis diagnosis and research. MiR-15b-5p and miR-378a-3p have been documented to be upregulated in platelet exosomes during sepsis, activating the Akt/mTOR-related autophagy pathway in neutrophils, resulting in overproduction of NET and exacerbating the severity of sepsis [120]. As a result, miR-15b-5p and miR-378a-3p have emerged as potential biomarkers for the diagnosis of sepsis and as therapeutic targets [120]. These discoveries greatly broaden the understanding of platelet roles beyond their traditional functions, underscoring their involvement in immune responses and potential as targets for sepsis diagnosis and treatment.

### 2.10. Exosomal miRNA form Bodily Fluids

In sepsis, the role of miRNAs is not limited to the exosomes secreted by various types of cells discussed above. Exosomes originating from different cells and directly released into bodily fluids such as plasma and serum also significantly influence intercellular communication and immune regulation. These exosomes not only mediate cell and organ dysfunction, influencing the progress of sepsis, but also serve as a remarkably stable source of disease biomarkers in bodily fluids [30, 121]. A particular study identified 25 differentially expressed miRNAs in the plasma of patients with sepsis compared to healthy individuals, among them, downregulation of miR-335-5p, miR-331-3p, and miR-301a-3p was identified as critical prognostic indicators, potentially affecting the progression of sepsis through the MAPK and PI3K-Akt pathways [27]. Furthermore, miR-1-3p, present in plasma exosomes, is upregulated in sepsis, leading to a reduction in the expression of stress-related endoplasmic reticulum protein 1 (SERP1), thus inducing endothelial cell damage and dysfunction, and contributing to the onset of sepsis [122]. Likewise, miR-210-3p is also found to be upregulated in plasma exosomes and targets ATG. This upregulation promotes the secretion of inflammatory factors in lung tissue, thus contributing to sepsis-induced acute lung injury [123]. miR-7-5p, which is also found to be upregulated in plasma exosomes in patients with sepsis, regulates the expression of the antiapoptotic gene Bcl-2, inhibiting T lymphocyte apoptosis and thus mitigating sepsis damage [124]. However, miR-885-5p, which is upregulated in serum exosomes, increases NF-*κ*B expression by reducing HMBOX1, promoting myocardial dysfunction in sepsis [125]. Furthermore, studies have shown significant upregulation of plasma exosome miRNAs, such as miR-126-3p, miR-122-5p, miR-146a-5p, miR-145-5p, miR-26a-5p, miR-150-5p, miR-222-3p, and miR-181a-5p, and these miRNAs are closely associated with inflammation [126]. Research involving cerebrospinal fluid revealed that during systemic inflammation, exosomes in the fluid secreted miR-146a, miR-155, miR-1a, and miR-9, all of which are upregulated and have been confirmed to originate from choroid plexus epithelial cells (CPE), offering new diagnostic avenues for sepsis [19]. These findings highlight that in sepsis, exosomes derived from immune cells and exosomal miRNAs undergo dynamic changes across a wide range of cells, present valuable diagnostic and therapeutic targets (Table 1).

### 2.11. Exosomal miRNA in Sepsis Treatment

In monotherapeutic miRNA approaches, due to the potential for a single miRNA to bind up to 200 target mRNAs—each possessing diverse functions including transcription factors, receptors, and many others—off-target effects and related toxic responses have been observed. Depending on the route of administration and intracellular delivery mechanisms, miRNA therapies may not be confined to the target tissues or cells, possibly leading to systemic side effects [128]. In an animal study, a miR-34a mimic was absorbed not only by tumor tissues but also by the bone marrow and spleen, resulting in severe adverse reactions [129]. Similarly, miR-34a was utilized in a Phase I clinical trial using a different delivery method for the treatment of advanced solid tumors. While the formulation did show promise, only three patients achieved partial remission, and 12 patients maintained stable disease. Surprisingly, 4 patients died due to severe adverse immune reactions, leading to the termination of the trial [130, 131]. In recent years, RNA-based therapies have been continuously evolving to minimize off-target effects and other adverse reactions. Chemical modifications of RNA; novel RNA constructs such as circular RNA, self-amplifying RNA, and gene editing tools hold significant potential [128]. However, challenges remain in terms of sensitivity, specificity, and the delivery process. The future of RNA-based therapies depends heavily on the development of targeted delivery mechanisms. Creating new and enhanced delivery systems is crucial for effectively directing miRNA to specific tissues or cells. This includes exploring the potential of exosome-mediated delivery as a promising avenue for precise therapeutic intervention.

Exosomes not only play a role in exacerbating sepsis-related pathological processes but also have the potential to resist injury. These exosomes, containing specific bioactive molecules, offer advantages over traditional cell therapy, including reduced immunogenicity, lower risk of tumor occurrence, and lack of matching ligands [132, 133, 134, 135]. Thus, exosomes have significant therapeutic potential in sepsis treatment and as drug delivery vehicles for various diseases [4, 136]. For example, exosomes derived from immature dendritic cells, containing MFGE8, can improve apoptotic cell clearance and inhibit the release of TNF-*α* and HMGB1, which reduces the acute systemic inflammatory response and mortality in sepsis [73]. miRNAs within exosomes can also be harnessed for sepsis treatment, either actively or passively loaded, to precisely regulate gene expression by interfering with mRNA translation [34]. Delivering exosome-related miR-126 to the right carotid artery in CLP mice significantly reduced the expression of VCAM-1 and ICAM-1, demonstrating an improvement in myocardial function in sepsis [137]. Exosomes released into the lumen by intestinal epithelial cells are believed to, through a paracrine mechanism, reach adjacent cells and carry miRNAs such as miR-19a, miR-21a, miR-27a, and miR-126a. These miRNAs play a functional role in negatively modulating the expression of pro-inflammatory cytokines [138]. Due to carrying specific biological information from the original cells, the exosome could perform the corresponding biological functions instead of cell therapy.

In recent years, the role of MSCs in the regulation of immune and inflammatory responses has received increasing attention. Therefore, the potential application of MSC-Exos in the treatment of sepsis has become a research hotspot. MiR-223 in MSC-Exos, for example, controls and inhibits inflammation by downregulating Sema3a and Stat3 transcription factors, and alleviates cardiac dysfunction in cecal ligation and puncture-induced sepsis in mice [139]. Human adipose MSCs-exosomes, transferring miR-145, inhibit MRP1 expression and mediate an increase in antibacterial activity through LTB 4/BLT1 signalling, showing potential in sepsis treatment [140]. Additionally, MSC-Exos overexpressing miR-30b-3p prevents ALI by downregulating SAA3 expression and inhibiting ACE cell apoptosis [141]. MiR-146a in MSC-Exos inhibits NF-*κ*B signalling pathway, reducing inflammation in inflammatory diseases [142], while miR-548e-5p and miR-146a-5p reduce LPS-induced inflammation [143]. Human umbilical cord MSC-Exos, upregulating miR-146b, reduce IRAK1 expression and inhibit NF-*κ*B activity, thus improving acute kidney injury and sepsis symptoms [144]. Moreover, miRNA in adipose stem cell-derived exosomes can also improve organ dysfunction in sepsis. For example, miR-342-5p upregulated in these exosomes targets HK-2 cells and inhibits TLR9, alleviating acute kidney injury in sepsis [145].

In recent investigations, the majority of miRNAs encapsulated in MSC-exos demonstrate cell-mediated activation, which significantly influences macrophage polarization. These miRNAs induce macrophages to produce elevated levels of anti-inflammatory cytokines while concurrently suppressing the production of pro-inflammatory cytokines. This modulation of cytokine profiles effectively mitigates inflammatory responses and ameliorates the symptoms of sepsis.

Although numerous studies have explored the role of miRNAs within exosomes, they frequently overlook the critical issue of functional dosage. Each miRNA operates at a distinct effective concentration. For instance, research on miR-17-92 has demonstrated that at a lower dosage (0.00003 *µ*g of plasmid), it reduces the viability of colorectal cancer cells, whereas at higher dosages (0.3 *µ*g of plasmid), it paradoxically enhances cell activity [146, 147] Additionally, within the milieu of extracellular vesicles, various miRNAs may compete or antagonize each other, underscoring the importance of precise dosing in exosomal therapies.

Furthermore, exosomes from donors at different stages of life demonstrate variations in sepsis treatment, which may be related to the internalization of exosomes and their miRNA content. MSC-Exos from aged and young donors differ in their roles in alleviating acute lung injury and macrophage polarization [148]. This underscores the heterogeneity and individual variability of exosomal miRNAs, which present formidable challenges in clinical research. These challenges are exacerbated by the dosage-dependent differential effects observed within exosomal contents.

Moreover, to further minimize off-target effects and enhance the therapeutic efficacy and safety of exosomal miRNA, treatments advancements in MSC research have extended beyond their natural phenotypes. Genetically modified MSCs, designed to augment therapeutic efficacy, exhibit modified exosomal profiles, particularly in terms of miRNA content due to specific genetic alterations. Exosomes derived from transgenic MSC lines overexpressing hypoxia-inducible factor 1-*α* (HIF-1*α*) and telomerase demonstrated enhanced effectiveness in suppressing the proliferation of activated T cells compared to those from unmodified MSCs [149]. These modifications lead to exosomes with significantly enhanced therapeutic properties, including controlled miRNA concentrations, thus providing promising avenues for advanced sepsis treatment strategies [146, 150]. Surprisingly, there is an ongoing project involving the use of transgenic mesenchymal stem cells for the treatment of sepsis (NCT04961658). In addition to these concerns, the therapeutic application of exosomal miRNAs faces several formidable challenges that must be addressed to enhance their clinical utility. Despite the promising molecular delivery capabilities of exosomes and the regulatory potential of miRNAs, significant obstacles in delivery efficiency, clinical translation, and safety persist. The absence of standardized protocols for exosome isolation and purification further complicates these issues. Addressing these challenges is imperative to optimize the therapeutic efficacy and reliability of exosomal miRNA treatments. Comprehensive research is necessary to elucidate their mechanisms thoroughly, confirm their clinical safety and efficacy, and foster the development of exosomal miRNA-based treatments for sepsis (Table 2).

## 3. Conclusions

Exosomes, which originate from various cell types, act as mirrors that reflect the functions of their source cells, playing a pivotal role in key biological processes associated with sepsis. Exosomal miRNAs are involved in the onset and progression of sepsis and have potential clinical value in the treatment of sepsis. These miRNAs exert dual effects, promoting or mitigating inflammatory responses, influencing coagulation mechanisms, modulating endothelial function, and dictating organ protection or damage. This dichotomous nature provides valuable information for understanding disease progression and developing novel therapeutic strategies. The stability and detectability of these small molecules in bodily fluids make them ideal for noninvasive diagnostic applications.

Although the potential of exosomal miRNAs in the diagnosis and treatment of sepsis is promising, research in this field remains in its infancy. To date, no clinical trials have been conducted on exosomal miRNA treatment for sepsis.

Future research could lead to more precise diagnostic and therapeutic strategies, potentially involving the use of engineered exosomes. These engineered exosomes could serve as components in innovative vaccine designs or drug delivery systems, facilitating precise and effective targeting of lesions for more personalized exosomal miRNA-based treatments. Furthermore, a deeper understanding of sepsis mechanisms is expected to enhance our knowledge of exosomal miRNA roles, thereby guiding more effective clinical applications.

The complex relationship between exosomal contents from various cell types and the progression of sepsis remains largely unexplored. In addition to noncoding RNA, other exosomal components, such as the eCIRP membrane protein released by macrophages during sepsis, play a significant role. This protein, which promotes the production of cytokines and neutrophil migration, exacerbating inflammation, has emerged as a potential target for the diagnosis of sepsis and the suppression of inflammation [158]. The relationship between exosomal membrane proteins and the development of sepsis represents a novel area of research. There is an urgent need to identify targets in early-stage sepsis exosomes, to determine their diagnostic accuracy and therapeutic value, facilitating their effective translation into clinical practice.

In conclusion, a comprehensive understanding of the diversity and functions of exosomal miRNAs, along with the application of expanding omics methodologies to exosomes, opens new avenues in the diagnosis, treatment, and prevention of sepsis. This burgeoning field promises significant advances in clinical practice soon.

## Figures and Tables

**Table 1:** Exosomal miRNAs that accelerate the onset and progression of sepsis.

Origin	Cargo (miRNA)	Form	Targets	Function	Signal passage	Implication	Ref.
Neutrophils	miR-30d-5p	Upregulate	M1	Induces M1 macrophage polarization and triggers macrophage pyroptosis	NF-*κ*B signalling pathway	Promote lung inflammation in sepsis by inducing macrophage M1 polarization and triggering macrophage pyroptosis	[48]
Neutrophils	miR-142-3p and miR-451	Upregulate	Endothelial cells	—	—	Induces endothelial cell damage and inflammatory cascades	[47]
Neutrophils	miR-150-5P	Upregulate	—	—	—	Plays an important role in the progression from sepsis to cardiomyopathy	[49]
Monocytes	miR-155 and miR-223	Upregulate	Endothelial cells	Activate TLR4/NF-*κ*B signalling pathway	TLR4/NF-*κ*B signalling pathway	Increase the expression of inflammatory factors, aggravates the inflammatory response and endothelial damage	[63]
Macrophages	miR-21-5p	Upregulate	—	—	—	Regulates the occurrence of inflammation	[64]
Dendritic cells	miR-155	Upregulate	—	—	—	Enhances inflammatory response to endotoxin	[74]
Lung epithelial cells	miR-92a-3p	Upregulate	Macrophages	Induction of alveolar macrophage activation in septic lung injury	NF-*κ*B signalling pathway	Activating alveolar macrophages and increasing ischemia-reperfusion-induced inflammation	[106]
Epithelial cells	miR-320a and miR-221	Upregulate	Macrophages	—	—	Increased macrophage-mediated inflammatory response	[107]
Renal epithelial cells	miR-19b-3p	Upregulate	—	—	—	Increase the activation of M1 macrophages and aggravate kidney damage	[20]
Endothelial cells	miR-99a b	Upregulate	monocytes	Enhance the pro-inflammatory effect of monocytes	NF-*κ*B signalling pathway	Promote the pro-inflammatory response of monocytes by targeting the expression of mTOR	[99]
Platelets	miR-15b-5p and miR-378a-3p	Upregulate	PMN	Regulate PMN, thereby promoting NET formation	PI3K/Akt/mTOR signalling pathway	Exacerbating NET formation and aggravating the occurrence of septic shock	[120]
Plasma	miR-1-3p	Upregulate	Endothelial cells	Reduce the expression of the target gene SERP1	Wnt/*β*-catenin or NF-*κ*B signalling pathway	Inhibits proliferation and increases apoptosis, shrinking the cytoskeleton, thus inducing endothelial cell damage and dysfunction and promote the development of sepsis	[122]
Plasma	miR-1298-5p	Upregulate	SOCS6	Enhance cell permeability and induce inflammatory response	STAT2 signalling pathway	Causes damage to human lung bronchial epithelial cells and induces septic lung injury	[127]
Plasma	miR-210-3p	Upregulate	—	Targeted inhibition of ATG7 and autophagy-related protein expression	—	Promote cell apoptosis, increase tissue permeability, stimulate the secretion of inflammatory factors in lung tissue, and promote sepsis-induced acute lung injury	[123]
Serum	miR-885-5p	Upregulate	HMBOX1	Negative regulation of HMBOX1	NF-*κ*B signalling pathway	Increases inflammation such as IL-1*β* and IL-18, and promotes myocardial cell pyroptosis in sepsis	[125]
Serum (partly derived from alveolar epithelial cells)	miR-155	Upregulate	Macrophages	Promotes macrophage proliferation and inflammation by downregulating SHIP1 and SOCS1.	NF-*κ*B signalling pathway	Promote macrophage proliferation, induce TNF-*α* and IL-6 production, and promote septic lung injury	[16]

M1, M1 macrophages; NF-*κ*B, nuclear factor-*κ* B; TLR4, toll-like receptor; PMN, polymorphonuclear leukocytes; NET, neutrophil extracellular traps; PI3K, phosphoinositide 3-kinase; Akt, Protein kinase B; mTOR, mammalian target of rapamycin; SERP1, stress-related endoplasmic reticulum protein 1; SOCS6, suppressor of cytokine signaling 6; STAT2, signal transducer and activator of transcription 2; HMBOX1, histone methyltransferase and nucleosome binding protein 1; IL, interleukin; SHIP1, Src homology 2 domain-containing inositol polyphosphate 5-phosphatase 1; SOCS1, suppressor of cytokine Signaling 1; TNF, tumor necrosis factor.

**Table 2:** Exosomal miRNA plays a protective role in the development of sepsis.

Origin	Cargo (miRNA)	Form	Targets	Function	Signal passage	Implication	Ref.
ADSCs	miR-146a	Upregulate	Macrophages	Involved in *β*MSC-exo-mediated M2 polarization *in vitro*	IRAK1, TRAF6, and IRF5 signalling cascade	Transferred into macrophages and induced downregulation of M1 markers and upregulation of M2 markers	[142]
ADSCs	miR-342-5p	Upregulate	HK-2 cells	Inhibit TLR9	—	Entering HK-2 cells and attenuating acute kidney injury in sepsis by inhibiting TLR9	[145]
ADSCs	miR-21	Upregulate	Macrophages	Targeted inhibition of PDCD4 transcription and expression	—	Promotes macrophage polarization to the M2 phenotype, thereby alleviating sepsis	[151]
MSCs	miR-30b-30p	Upregulate	—	—	—	Inhibit the apoptosis of type II alveolar epithelial cells and improve acute lung injury	[141]
MSCs	miR-27b	Upregulate	Macrophages	JMJD3 expression in macrophages	NF-*κ*B signalling pathway	Inhibit the pro-inflammatory effects of BMDM, thus controlling the development of sepsis	[152]
MSCs	miR-26a-5p	Upregulate	MALAT1	Inhibit MALAT1	—	Inhibiting MALAT1 and MALAT1-enhanced oxidative stress may effectively prevent sepsis-induced acute liver injury	[153]
MSCs	miR-146a-5p	Upregulate	MYBL1	Targeted inhibition of MYBL1 expression	—	Promote LPS-induced cardiomyocyte proliferation and inhibit cell apoptosis	[154]
MSCs	miR-223	Upregulate	Macrophages and cardiomyocytes	—	Sema3A/Stat3 axis	Leads to suppression of macrophage inflammatory responses and attenuation of cardiomyocyte death during sepsis	[139]
BMMSCs	miR-141	Upregulate	PTEN	Targeted inhibition of PTEN	*β*-Catenin signalling pathway	Enhanced *β*-catenin activity to improve myocardial damage in septic mice	[155]
BMMSCs	miR-150-3p	Upregulate	INHBA	Promotes anti-inflammatory polarization of alveolar macrophages	—	Inhibits the pro-inflammatory polarization of alveolar macrophages in sepsis and alleviates LPS-induced sepsis	[156]
BMMSCs	miR-191	Upregulate	THP-1	Binds to and inhibits expression of the 30-UTR of DAPK1 mRNA	—	Acts on THP-1 macrophages and inhibits LPS-induced inflammatory activation	[89]
BMMSCs	miR-21a-p	Upregulate	TLR4 and PDCD4	—	—	Targeting toll-like receptor 4 and programmed cell death 4 to suppress inflammation and suppress sepsis	[90]
Umbilical cord adipose stem cells	miR-146b	Upregulate	IRAK1	Reduce IRAK1 expression and inhibit NF-*κ*B activity	NF-*κ*B	Inhibiting the activity of NF-*κ*B alleviates acute kidney injury and reduces mortality	[144]
ADSCs	miR-145	Upregulate	—	Inhibit MRP1 expression	LTB4/BLT1 signalling pathway	Increased antibacterial activity, improved sepsis	[140]
Umbilical cord adipose stem cells	miR-548e-5p miR-146a-5p	Upregulate	—	—	—	Anti-inflammatory effect on human trophoblast cells	[143]
Lung Epithelial cells	miR-145	Downgrade	TGFBR2	Inhibits TGF-*β*/Smad signalling pathway	TGF-*β*/Smad signalling pathway	Inhibits LPS-induced inflammation and sepsis-induced lung injury	[108]
Endothelial cells	miR-125b-5p	Upregulate	DNA-TOP2A	Targeted inhibition of TOP2A expression	—	Preventing sepsis-induced acute lung injury by inhibiting TOP2A expression	[100]
Endothelial progenitor cells	miR-21-5p	Upregulate	RUNX1	Downregulation of RUNX1	—	Alleviating sepsis-induced kidney injury by downregulating RUNX1	[101]
Endothelial progenitor cells	miR-382-3p	Upregulate	BTRC	Targeted inhibition of BTRC expression	—	Targeted inhibition of BTRC expression and reduction of I*κ*B*α*/NF-*κ*B phosphorylation, thus alleviating CLP-induced organ damage and immunosuppression in septic mice	[102]
Endothelial progenitor cells	miR-126	Upregulate	—	—	—	Improve acute lung injury caused by sepsis	[88]
Macrophages	miR-21-3p, miR-146a and miR-146b	Upregulate	—	—	—	Prevent overactivation of innate immune response	[59]
M2	miR-216a	Upregulate	HMGB216	Negatively regulates HMGB216 expression	TLR4-NF-*κ*B signalling pathway	Serum inflammatory factors are significantly reduced, and the ability to resist injury is improved.	[61]
M2	miR-24-3p	Upregulate	Tnfsf10	Targeted inhibition of TNFSF10 expression	—	Enhanced myocardial protection in sepsis	[62]
Dendritic cells	miR-146a	Downgrade	—	—	—	Inhibit endotoxin inflammatory response	[74]
Serum	miR-21	Upregulate	—	Inhibited the expression of PDCD-4 and PTEN	NF-*κ*B and PTEN signalling pathways	Downregulates cleaved Caspase-3 expression, thus reducing inflammation and apoptosis, and attenuating sepsis-induced renal injury	[157]
Plasma	miR-7-5p	Downgrade	proapoptotic gene Bad	the expression of proapoptotic gene Bad	—	Inhibiting T lymphocyte apoptosis reduces sepsis mortality	[124]

ADSCs, adipose-derived stem cells; exo, exosome; RAK1, receptor-interacting protein kinase 1; TRAF6, tumor necrosis factor receptor-associated factor 6; IRF5, interferon regulatory factor 5; M1, M1 macrophages; M2, M2 macrophages; HK-2, human kidney-2; TLR, toll-like receptors; PDCD4, programmed cell death protein 4; JMJD3, jumonji domain-containing protein 3; BMDM, bone marrow-derived macrophages; MALAT1, metastasis-associated lung adenocarcinoma transcript 1; MYBL1, myb proto-oncogene-like 1; Sema3A/Stat3, semaphorin 3A and signal transducer and activator of transcription 3; BMMSCs, bone marrow-derived mesenchymal stem cells; PTEN,, phosphatase and tensin homolog; INHBA, inhibin subunit *β* A; LPS, lipopolysaccharide; THP-1, tamm-horsfall protein 1; 30-UTR of DAPK1 mRNA, 30 untranslated region of death-associated protein kinase 1 mRNA; IRAK1, interleukin-1 receptor-associated kinase 1; NF-*κ*B, nuclear factor-*κ* B; MRP1, multidrug resistance-associated protein 1; LTB4, leukotriene B4; BLT1, leukotriene B4 receptor 1; TGFBR2, transforming growth factor *β* receptor 2; DNA-TOP2A, DNA topoisomerase 2-*α*; RUNX1, runt-related transcription factor 1; BTRC, *β*-transducin repeat containing E3 ubiquitin protein ligase; CLP, cecal ligation and puncture; TNFSF10, tumor necrosis factor ligand superfamily member 10.

## Data Availability

This review article relies on previously published data, fully referenced within the text. No new data were generated during the course of this review.
